# The Role of Digital Technologies to Promote Collaborative Creativity in Language Education

**DOI:** 10.3389/fpsyg.2022.828981

**Published:** 2022-02-09

**Authors:** Moisés Selfa-Sastre, Manoli Pifarré, Andreea Cujba, Laia Cutillas, Enric Falguera

**Affiliations:** ^1^Department of Specific Didactics, Universitat de Lleida, Lleida, Spain; ^2^Department of Psychology, Universitat de Lleida, Lleida, Spain

**Keywords:** creativity, collaboration, technology, language, education

## Abstract

The importance of cultivating creativity in language education has been widely acknowledged in the academic literature. In this respect, digital technologies can play a key role in achieving this endeavour. The socio-cultural conceptualization of creativity stresses the role of communication, collaboration and dialogical interaction of creative expression in language education. The objective of this paper is to study the literature focusing on cases of collaborative creativity and technology embedded in language education. To this end, we carry out a systematic revision of state-of-the-art literature consisting of 26 blind peer-reviewed empirical studies selected from several databases that address our main research question, namely, which specific roles and forms of digital technology can be identified in the existing literature that support collaborative creativity in language education. Results show that the features of digital technology unfold a range of learning opportunities in language education and can play three different roles in promoting collaborative creativity: (1) as a tutoring device that guides the implementation of key co-creation skills; (2) as a tool that enables and shapes the development of co-creative thinking skills; and (3) as a medium that creates rich and resourceful environments to stimulate the emergence of collective creative processes. The paper also reveals that these three roles can be performed using a wide range of interactive technologies that encourage students to participate in a rich, co-creative language learning experience and equip learners with key competences to approach complex problems in a globalised and hyper-connected world. Finally, this paper may contribute to developing future language technology-enhanced learning projects capable of promoting key collaborative and creative processes.

## Introduction

Creativity has been identified as the backbone of the skills needed to participate fully in the 21st century society, both in professional and everyday situations that require innovative responses. Also, creativity is regarded as one of the key competences to approach complex problems in a globalised and hyper-connected world (Gretter and Yadav, 2016; Henriksen et al., 2016). Consequently, over the last decade and globally, educational policies have been implemented aimed at including in curricula competences and contents that promote creativity and innovation (Van de Oudeweetering and Voogt, 2018).

Recent educational research agrees that creativity is the ability to generate novel, appropriate and valuable ideas that can lead to producing original and valuable products or learning outcomes (Rojo, 2019). Therefore, the definition of creativity includes two characteristics: on the one hand *originality* refers to novelty, infrequency and uniqueness; on the other hand *usefulness refers* to utility, appropriateness, fitness or valuableness for the community (Hernández-Torrano and Ibrayeva, 2020).

Recent trends consider creative acts to be socio-cultural in nature and origin. From this perspective, the actions aiming to generate original and valuable products are seen as a social, rather than as an individual phenomenon. Since cultural traditions, social practices and social artefacts regulate and transform the human mind (Shweder and Sullivan, 1990; Glăveanu, 2018), there is a profound interdependence between individuals and their socio-cultural context. The transactions, interactions and activities between these two systems are at the root of creativity. According to Vygotsky et al. (1996), context-mediated action is the socio-cultural genesis of mental functions. Thus, the socio-cultural conceptualisation of creativity emphasises the role of intersubjectivity, communication and dialogical interaction in creative expression (Glăveanu, 2010). There is no doubt that creativity emerges from the close and binding relationship between language and thought.

The focus of educational research on socio-cultural aspects of creativity has led to the coinage of such concepts as *collaborative creativity*, *collaborative creativity*, *group creativity* or *distributed creativity* (Sawyer, 2012; Glăveanu, 2014). Creativity has been defined as a social process (Sawyer, 2012) that emerges and develops among group situations. Indeed, great innovations are often the result of group work, and social judgement and communication play an important role in developing creative products (Glăveanu, 2018). In this respect, collaborative creativity can be considered as the emergence of shared ideas between two or more individuals (Sakr, 2018). Also, Sun et al. (2022) highlight that group processes, such as sharing, negotiation, group communication and interaction processes, are decisive factors of collaborative creativity. Tanggaard (2020) claims that the situated, social nature of creative practices requires a basic dimension of *togetherness* because we create with the support and engagement of others, and the support of tools and artefacts created by former generations. Sakr (2018) claims the importance of taking into consideration the affective dimensions of collaborative creativity. In this paper, we aim to explore how collaborative creativity supported with technology is promoted in language education.

In the specific case of language education, creativity processes emerge when there is a requirement to meet the challenges posed by language teaching. In this article, collaborative creativity in language education is characterised as the collaborative construction of an original and valuable product that gives a creative answer to language learning challenges. The difference between solving these problems individually or collaboratively is abysmal. While in the former, there is a univocal and unidirectional dialogue, in the latter, i.e. when two or more subjects are involved in the creation of creative thinking, productivity and collaboration between equals is fostered. This results in rich communication of experiences in pursuit of a common good: in other words, ‘students are engaged in higher level thinking activities such as problem solving and discussion of complex ideas’ (Shuler et al., 2010, p. 11). There is, therefore, an engagement between people to solve what Montalvo (2011) calls *linguistic enigmas*. In this scoping review, we only take into consideration those pieces of research that promote creativity in collaborative environments.

Nowadays, we witness the rapid development of digital and interactive technologies connecting people in multiuser working spaces where users can interact, share and externalise their ideas in open spaces, interplaying with others’ voices in different and multiple multimodal channels. As a result of this active online dialogue, new, dynamic and co-created knowledge can emerge (Pifarré, 2019). On this issue, Wegerif (2015) argues that technology shapes human thinking and impacts on how we think and interact with others. Therefore, technology can play an important role in mediating students’ creative actions as well as engaging them into meaning-making and collaborative knowledge creation (Säljö, 1999). Also, Mercer et al. (2019) state that we think *with* and *through* artefacts that constitute mediational means endowed with affordances and constraints.

Previous research has characterised distinct features of interactive technology that can play a role in resourcing, promoting and shaping co-creative dialogues (Major et al., 2018). Certainly, digital technologies open up new possibilities for creating and visualising the links between language and thought that allow for a multimodal representation of creative ideas. Ntelioglou et al. (2014) emphasise that the multimodal interaction of technologies facilitates 21st century education in that it promotes broader literacy beyond simple literacy skills by incorporating multiple modes of meaning-making and communication (e.g. auditory, visual, linguistic, spatial and body modes) on the one hand; on the other, the multimodal interaction of technologies provides pedagogical support for learners to optimise their language and literacy learning. For example, digital storytelling has been used to create collaborative storeys as well as to favour language learning specially, learning an L2 in multilingual learning (Anderson et al., 2018; Andayani, 2019; Tyrou, 2021).

Technology can play a crucial role in solving language learning problems. In fact, there is a growing application of digital and interactive technologies to language education. Our interest focuses not only on learning what types of digital environments have been mostly used to stimulate collaborative creativity in language education, but also on reviewing how technology has been used to foster collaborative creativity processes and what results have been obtained from its use.

Our review paper aims to fill this research gap and provide new knowledge on how research in the field of language education has used technology to promote collaborative creativity processes. In this line, identifying the most salient features of existing research may provide an insight into further research on new pedagogies involving creativity in language education with the use of digital technologies.

## The Study

Over the last decade, language education has gradually incorporated the use of a wide range of interactive mobile technology widely used in scientific research. The introduction of such technology in language classrooms has generated opportunities and challenges in the design of learning scenarios that promote collaborative creativity competences. Surprisingly, there are no review studies that analyse the role of technology in supporting and organising collaborative and creative processes in the teaching and learning of language content. Such studies could provide valuable knowledge for outlining theoretical frameworks and pedagogical guidelines to better design language teaching and learning projects that use technology to promote creativity.

To fill this research gap, this article offers a review of educational studies that combine the use of pedagogy and digital technologies to cultivate collaborative creativity competencies in language teaching and learning. This review focuses on research conducted at compulsory and post-compulsory education levels during 2008–2021, as will be seen in the Results and discussion sections.

Our research question is ‘Which specific roles and forms of digital technology can be identified in the existing literature that support collaborative creativity in language education?’

## Materials and Methods

### Bibliographical Search and Criteria for the Selection of Studies

We carried out a scoping review to identify the most relevant studies on the development of collaborative creativity in language education by means of digital technology. This systematic review aims to gather evidence on the role of digital technology in promoting social creativity in language education and carry out a quantitative and qualitative analysis. Our analysis aims to identify and evaluate existing studies rather than use statistical techniques (e.g. metanalysis) that combine results of these studies to obtain global measuring parameters. Our methodological framework follows the PRISMA statement, a recent guide of systematic reviews launched by Page et al. (2021). The databases consulted are Web of Science (WoS), Scopus and Google Scholar, as they are the most widely acknowledged sources of reference to obtain updated systematic reviews (Codina, 2018: 30–33). We carried out searches in these databases through the advanced search function entering the following keywords and phrases: ‘language education’, ‘creativ*’, ‘collab*’, ‘techno*’ and ‘learning’.

On the first search attempt, we realised that the word ‘technology’ could be restrictive as some articles used keywords, such as ‘computer’, ‘digital’ or ‘video’. In view of this, we decided to replace the word ‘technology’ with the keywords: ‘comput*’, ‘digital’, ‘Web’, ‘video’, ‘blog’, ‘Wiki’ and ‘podcast’. As a result, we managed to collect an appropriate selection of studies for our research. It should be noted that our search covered the last 13 years (from 2008 to 2021), as during these years, there has been an increased integration of technology in language teaching and learning classrooms. In this respect, creativity and collaboration among peers in teaching and learning flourish when learners need to give a novel and original reply to the group.

We established inclusion and exclusion source registers for the systematic review. They are indicated in Table 1.

**Table 1:** Inclusion and exclusion criteria.

Inclusion criteria	Exclusion criteria
Publications were included if they report on collaborative creativity processes with technology	Conference proceedings as we focused on blind p*eer-reviewed* publications
Publications that focus on teaching and learning processes in language education	Books and book chapters were excluded because of accessibility difficulties
Publications that were peer-reviewed	Publications that were not written in Spanish or English
Publications focused on primary and secondary education as well as university studies	
Papers published between 2008–2021	

**Figure 1:**
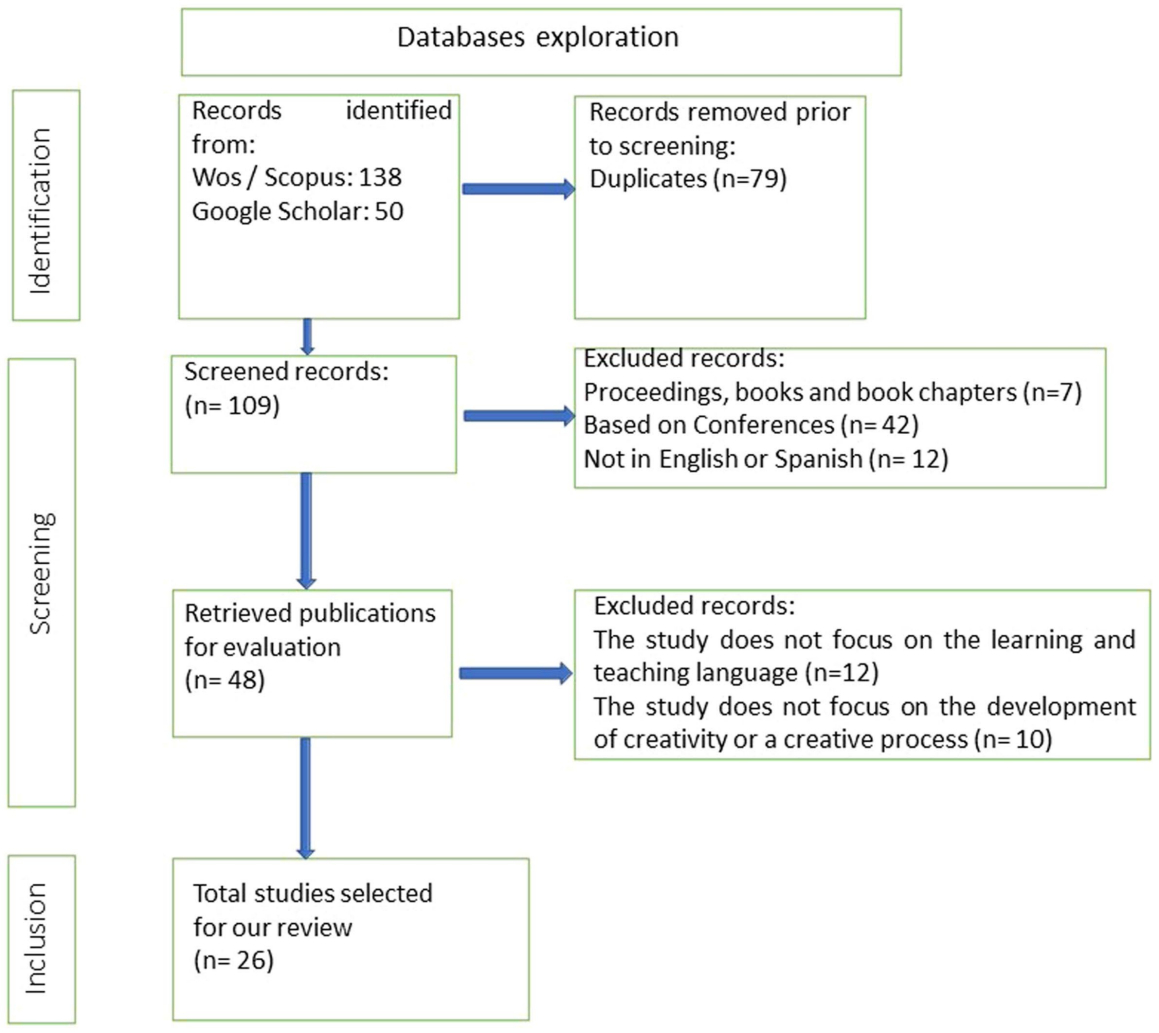
Screening procedure of the papers.

Two independent reviewers conducted all stages of study selection for this review; discrepancies in papers that partially met the criteria were re-examined by a third reviewer and resolved by consensus. Our initial pool was 188 articles, of which 79 were duplicates as they appeared in all three databases consulted. Through the first screening, we discarded the following three types of documents: (a) studies from conferences and/or communications: only blind peer-reviewed articles were considered; (b) books and book chapters were discarded due to the difficulties of having open access to these documents; and (c) articles that were not written in English or Spanish. Figure 1 summarises the screening procedure and the inclusion and exclusion criteria for the paper review selection.

After this first search, 48 scientific articles were read and assessed for their suitability to achieve the objectives of our research. From these, 12 of them were discarded as they were not directly related to the domain of language teaching and learning. In addition, 10 more articles did not directly discuss research linked to the development of collaborative creativity in language teaching and learning processes. Finally, we narrowed down a selection of 26 relevant studies in line with the research question of this study. A description of all the selected papers is shown in Table 2.

**Table 2:** Summary of review studies reporting on collaborative creativity practices with technology in the teaching and learning of languages.

Authors	Technology Role	Technology Type	Creative processes	Main results
Armstrong and Retterer, 2008	Medium	Blog	Collaborative writing in a blog, as a second language learning (Spanish)	Students who have written the longest texts have improved their oral expression in Spanish After participating in the blog writing experience, 100% of the students feel more confident when writing in Spanish
Lund and Rasmussen, 2008	Medium	Wiki	Collaborative writing to produce wiki content that describes a typical British town Creativity: the invention of a city and the aspect of its presentation in the wiki	During the project development, the students have gone through writing processes as interpretation, construction and reconstruction Coordination with group members has created a group task identity and has made students commit to the task
Mak and Coniam, 2008	Medium	Wiki	Collaborative writing to produce wiki content that describes the different facilities and features of their school. Creativity: the aspect of the brochure	Using the wiki as an online collaborative writing environment, students have improved their written expression skills: expanding, rearranging and correcting texts
Rojas-Drummond et al., 2008	Medium	Word processing	Co-construction of texts and multimedia products	Establishment of intertextual and intercontextual relations between texts through the use of ICTs Development of dialogic and textual production strategies Appropriation of diverse cultural artefacts for the construction of knowledge
Yang and Huang, 2008	Medium	Web and Apps	Sharing oral and written productions among peers to learn an L2	Difficulty of integrating technology into the L2 classroom at initial levels of L2 teaching and learning Proposals for concrete integration of technology in the L2 classroom
Comas-Quinn et al., 2009	Tool	Mobile phones Blog	Creation of digital texts to learn about an L2 and its culture	Acquisition of the linguistic and cultural components of an L2 through the creation of digital texts and narratives
Kukulska-Hulme, 2009	Tool	Mobile phones	Mobile learning as a new way to learn L2	The mobile device facilitated the communication between students and teacher to learn the meaning of words, outside the classroom
Lee, 2009	Tool	Blog Podcast (Audacity, iMovie)	Collaborative writing to enhance cultural awareness	This article reports a Spanish–American telecollaborative project through which students created blogs and podcasts for intercultural exchanges in light of socio-cultural perspectives
Kilickaya, 2010	Medium	Audio and video platform *Xtranormal*	Creating animated films with voice-over audio	Generation of listening contexts to improve writing, reading and pronunciation of an L2
Montalvo, 2011	Tutor	YouTube Blog	Creative Multimedia Riddles	Improved association of ideas, analysis of metaphors and discovery of analogies Distinct cognitive and sensory experience in multimedia riddle interaction
Akinwamide and Adedara, 2012	Medium	Digital audio and radio platforms	Digitize pedagogy in language teaching and learning	Digitize language learning, to facilitate its study outside the classroom Examples of technological tools as a means of language learning
Contreras Salas, 2012	Tutor	Web 2.0 Social media Wiki Blog Podcast *Folksomies*	Organising social networks. Sharing videos and photos. Creating wikis	Encouraging creative and participatory work using ICT Continuous review of methodological guidelines for teaching English
Lorenzo et al., 2013	Medium	*MMOL* (*Massively Multiuser Online Learning*)	Development of microcontent immersed in collaborative virtual environments	The use of MMOL for second language learning shows that collaboration in a 3D educational context, in combination with the use of communication tools (chat, video chat or VoIP) and intelligent assistants (chatbots or NPCs), has a positive effect on the individual acquisition of language content
Mellati and Khademi, 2014	Tool	Web based (*Net, Email*)	Write an essay in a digital environment and receive and give feedback to peers	Encouraging writing in a digital environment Promotion of E-collaboration and collaborative work: give feedback to classmates’ essays
Ntelioglou et al., 2014	Tool	Power Point iMovie iPhoto	Descriptive narratives	High quality of texts More participatory and multimodal learning dynamics. Connection to learners’ lives
Mellati and Khademi, 2015	Tool	WhatsApp	Collaborative writing using WhatsApp	The use of the application facilitates a more colloquial expression, which gives a sense of belonging to the group
Stevenson et al., 2015	Tool	*Storyboards*	Creative linguistic activity	Generating and representing creative ideas for storytelling Technology facilitates the participation of diverse members and the development of multimodal literacy
Cruz and Orange, 2016	Tutor	WebQuest Gloster Kahoot	Creative oral presentations	Technology mentors key creative processes
Naqvi and Al Mahrooqi, 2016	Tool	Digital video	Production of an audiovisual message and write a digital text about a commercial product	The use of digital tools in collaborative and creative environments improves motivation for second language learning
Anderson et al., 2018	Medium	Multilingual Digital Storytelling (MDST)	Multilingual digital writing for multiliteracy development	It demonstrates the importance of an integrated and inclusive approach to languages in the framework of multilingualism
Schmoelz, 2018	Medium	Digital Storytelling (DST)	Creation of digital narratives to work and study the co-creativity	The work with digital narratives encourages co-creativity with a greater emotional involvement of the students and a greater commitment and control over the activity and the final results
Andayani, 2019	Tool	Digital storytelling using Microsoft Power Point or Microsoft Video Maker	Digital storytelling projects created by English student teachers for young learners of English as Foreign Language (EFL)	The use of digital narratives has several benefits for students: integration of technology, implementation of pedagogical theories, increased motivation, reduced anxiety about public speaking, and enhanced creativity
Olivier, 2019	Medium	Short videos	Creation of short videos by students with content related to language learning	Videos can be used as a means to motivate students to critically interact with content and to collaborate with new technologies to learn a language
Chubko et al., 2020	Tool	Digital storytelling (DST)	Intervention with DST created by STEM teachers for students with English as L2	Digital narratives help to improve the learning of scientific content, through collaborative and creative environments, by students of English as L2, at the same level as students who have it as L1
Yang and Yeh, 2021	Tool	Making videos (*YouTube*)	Production of videos to learn about the cultural component of a language	Making promotional videos on YouTube helped English students as a foreign language to be prepared to become socio-cultural agents to introduce themselves and their local culture to the world
Tyrou, 2021	Tool	Wikis	Wiki-mediated L2 collaborative writing	Collaborative writing through technology promotes, among other things, the improvement of: revision of texts (self-correction and peer correction), collaboration, knowledge of foreign cultures and languages

We then categorised the 26 studies as they constituted our research basis. After reading, checking and discussing the 26 selected papers, we agreed on the following: firstly, including the paper in one specific category or role of technology. Secondly, analysing how each type of technology was used in each paper to promote collaborative creativity. Thirdly, checking on the discrepancies solved using a consensus-based approach.

## Results

Table 2 provides an overview of the core data extracted from the selected studies. In order to identify the possible roles that technology could play in promoting students’ collaborative creativity in language education, we were inspired by the different ways of conceptualising the relationship between technology and teaching thinking and creativity developed by Loveless (2007) and Wegerif (2015). For the purposes of this study, as many as three different roles of technology in promoting students’ collaborative creativity in language education were identified as: (1) technology as a tutor that induces and models the execution of key co-creative processes for solving language challenges; (2) technology as a tool whose utilisation and appropriation of its characteristics by the students becomes an instrument to think creatively and collaboratively during language learning; and (3) technology as a medium or an environment that prompts the development of key collaboration and creativity processes.

**Figure 2:**
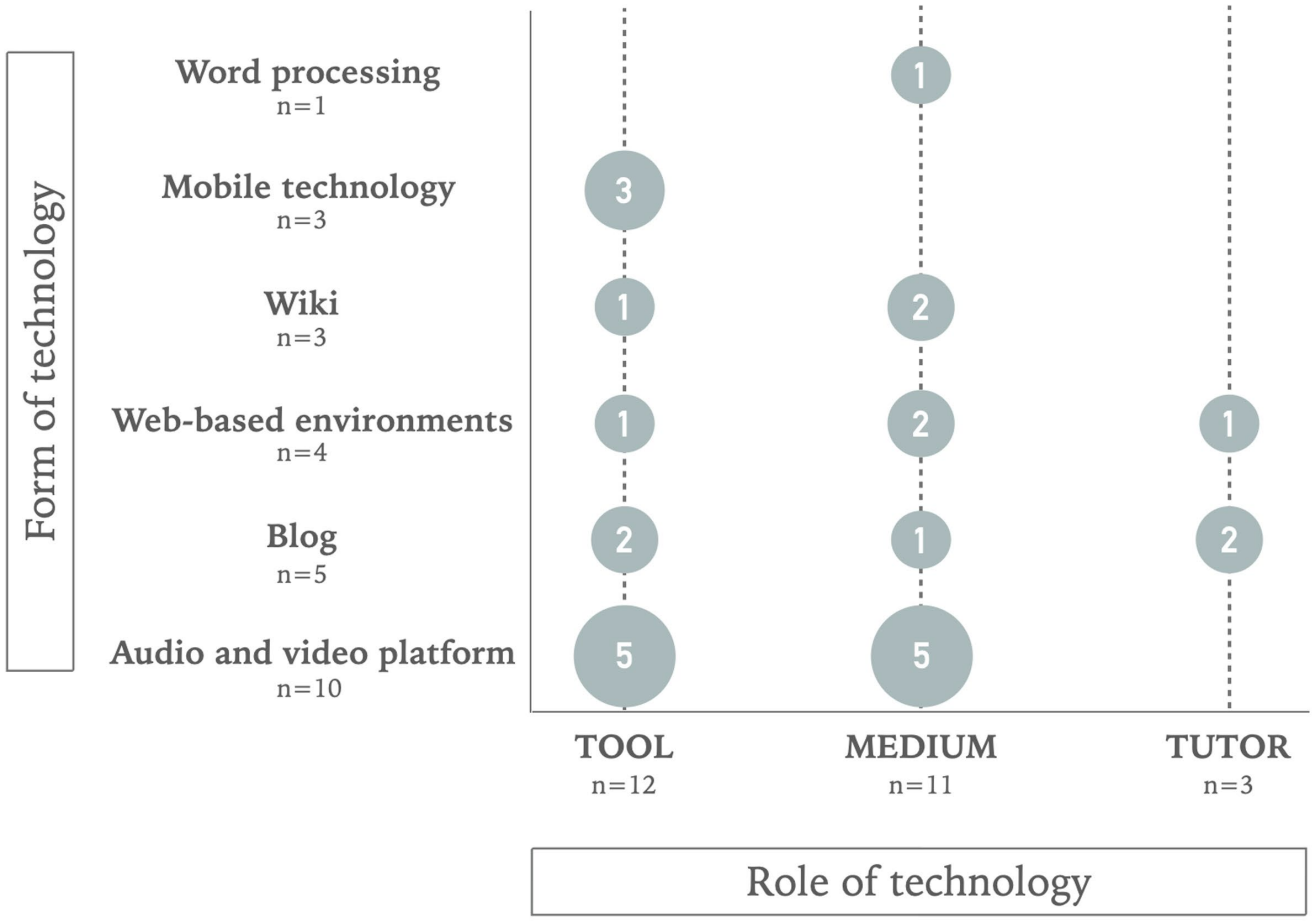
Form and role of the technology.

Figure 2 displays the results of the roles that technology plays in promoting students’ collaborative creativity in language education and the forms of technology used. As shown in Figure 2, technology as a tool is the most frequent role among the studies reviewed (*n* = 12). This role was introduced using a wide range of digital technologies. In any case, audio and video platforms are the most common forms of technology used as instruments that facilitate thinking co-creatively. Out of these 26 studies, 11 of them promoted the use of technology in language education as a medium that provokes co-creation. Fewer studies were found with a tutor role technology (*n* = 3).

Six forms of digital technology were identified in the studies reviewed in order to promote collaborative creativity in language education for all students. The studies analysed used mainly audio and video platforms (*n* = 10). A few studies used Blog (*n* = 5) and web-based environments (*n* = 4) and fewer studies used wiki (*n* = 3) or mobile technology (*n* = 3). Finally, a limited number of studies introduced word processing (*n* = 1). Audio and video platforms were the most frequent ones. When digital technologies were implemented as a tutor, Blog (*n* = 2) was the main one.

## Discussion

Recent research has shown that interactive technologies provide a set of tools than can enrich the learning context and nurture collaborative creativity processes (Henriksen et al., 2016). A specific technology imposes certain constraints, establishes preconditions for students’ behaviours and opens up a range of learning opportunities. Because of this, there is a need to analyse how technology is used to promote collaborative creativity in language education.

The qualitative analysis of the papers selected for this review identified three different roles of technology for promoting students’ collaborative creativity in language education, namely: tutor, tool and medium. In this section, we address the discussion of the results obtained in relation to these three different roles of technology.

### Technology as a Tutor of Co-creative Thought

Digital technologies can be seen as gadgets selected to guide a creative activity on the teaching and learning of a given linguistic content. From this point of view, technology can act as a tutor that encourages creative thinking by following pre-established guidelines and the design of scripts or prompts that promote the performance of specific creative skills. An example of this use in language learning is developed by Cruz and Orange (2016) and applied to Master studies. For their study, they use the multimedia poster *Gloster* to support and improve oral communication on a topic. In this study, technology promotes the development of key creative processes as it can increase opportunities to explore and play with materials, information and ideas around oral communication in a given language. The teacher often plays an important role in the use of technology, as it is the teacher who introduces technology in the classroom to teach curricular content creatively. However, the content-related use of technology can help raise awareness of the ways in which creativity relates to learning curricular knowledge.

In the study by Contreras Salas (2012), Web 2.0 tools are effective in creating information in collaboration with others, organising social networks, sharing videos and photos, and creating wikis, blogs, podcasts and *folksonomies*. The aim of their effective design was to offer a creative *virtual world* in which all the proposals related to the use of ICT were collected in a *Methodology Guide* as a tutorial guide for developing creative thinking. From this guide, teachers, in this case English ones, can draw different possibilities for the use of ICT which are not linear, but transversal and offer multiple possibilities of use and interaction. The result of this study showed that the inclusion of ICT in the teaching of English changed the dynamics of teaching in the learning of this language in 50% of the students. Indeed, students managed time asynchronously as participatory and collaborative work was necessary to carry out learning activities. On the other hand, teachers continuously reviewed the technological learning guidelines contained in the *Methodology Guide*. In this way, the learning process of the students was positively influenced by the methodological approaches of the language teacher.

Similarly, Montalvo (2011) focused on Peruan primary schoolchildren—years 4, 5 and 6. His study highlights the importance of collaborative learning in linguistic learning situations mediated by digital technologies. These technologies consist in audiovisual riddles which favour the exercise of creative thinking. Riddles represent a dialogical game between two or more people in which the riddle posed by the sender is tackled by the receivers, thereby establishing a dialogical game between the two. In order to ensure that audiovisual riddles are more widely accepted among new generations, the sender resorts to digital technology, giving them a digital treatment through YouTube. As Igarza (2009: p. 214) points out ‘YouTube is perhaps the Google of the next generation’. In addition to this tool, a blog recorded the response to each of the five riddles posed. Each of the riddles was based on an animated image and a text related to the content of that image. The solution to the task was carried out in groups of two, three or four students, which made it possible to observe the dynamics established among group members when working on the answer to a riddle. The conclusions of the study show that collaborative learning is one of the most functional ways of working in education. It is also closely related to what Montalvo (2011: p. 130) refers to as *collective intelligence*, today associated with Web 2.0 and social networks.

Creativity, peer collaboration and the use of technology have been, as shown above, the object of study, application and analysis in language teaching and learning environments, in which learners play an active role. However, as Contreras Salas (2012) points out in a study with Degree students of Elementary Education specialising in Humanities, Spanish and English of the Universidad Cooperativa de Colombia, based in Bucaramanga, it is necessary to encourage creativity using technology on teachers who have to teach language content. One of the technological avenues most widely explored in language teaching is Web 2.0, as it allows the teacher to share information and specific guides with the learner through the World Wide Web. Contreras Salas (2012) shows in his research how English teachers create Web 2.0 activities that offer different possibilities of working with ICT.

### Technology as a Tool That Facilitates Thinking Co-creatively

Socioculturalism argues that subjects learn to think by internalising the use of cultural tools, such as language or technology, which later become cognitive or critical thinking tools (Vygotsky, 1987). *Instrumental genesis* (Rabardel and Bourmaud, 2003) addresses the connection of human agents and technical artefacts through the concept of instrument. An instrument is a heterogeneous entity, composed of a technical artefact and a human agent. The instrument arises from a double developmental movement, which connects the artefact and its scheme of use. From this instrument, the agents interact and develop a creative product (Overdijk et al., 2012). Technology can thus be seen as a tool used to shape and develop an activity. Instrumentalization therefore changes the tool at the same time as it changes the subject using it.

Digital technologies offer different possibilities to solve language challenges creatively. Users can convert features of digital technologies into instruments for thinking that promote key collaborative and creative processes for solving a language activity.

The qualitative analysis of the papers selected for this review distinguished three different uses of digital technologies as instruments for promoting collaborative creativity actions in language education: (a) as a co-participation and engagement tool; (b) as a multimedia tool that enhances collaborative and creative writing strategies; and (c) a tool that supports linguistic thinking. Next, we will discuss these three uses of technology as a tool.

#### Technology as a Tool That Enhances Participation and Engagement of All Group Members to Jointly Create Knowledge

Engagement of all group members and being together is a basis of being creative (Tanggaard, 2020). Technology makes it possible to create and narrate a storey collaboratively by means of such tools as iMovie, iPhoto or digital storyboard (Stevenson et al., 2015). These are examples of the use of technology as a tool that facilitates creative linguistic activity of primary and secondary education students. This tool supports the participation of all group members to create joint knowledge and solve a complex creative task.

Kukulska-Hulme (2009) explores second language learning in playful digital environments that encourage peer interaction. This research shows the advantages of mobile learning to learn a second language. Taking the youngest generations as the basis of her research, the author shows how English as a second language (ESL) students develop peer-scaffolding strategies to communicate with each other and learn the meaning of new words with a mobile phone.

#### Multimedia Technology as a Tool That Supports and Generates Co-creative Writing Strategies

The process of writing a digital storey requires the implementation of new strategies related to generating, communicating and negotiating content in a meaningful way through multimedia information, which facilitates the development of literacy that includes aspects related to *doing* and *being* (Sawyer, 2012). Moreover, these new strategies developed while using technology favour the learning of processes associated with creativity, such as generation of ideas, their development and improvement, selection of the best ideas and their representation (Sun et al., 2022).

In a project with college English student teachers, Andayani (2019) describes the advantages of digital storytelling as a tool to promote collaborative learning skills when learning a second language. This study reports on the benefits in the learning of English of 31 pre-service students asked to create a digital fairy tale using multimedia and interactive technology.

The author concludes that the use of digital narratives has multiple benefits for future English teachers, such as the integration of technology, the implementation of pedagogical theories they had previously studied, the increase in motivation to finish the projects, the reduction of public speaking anxiety and, finally, the possibility of adding music and sound effects, which helped to better dramatise the storey. In post-project interviews, some of the study subjects state that the use of this tool fostered their creativity in designing English teaching and learning activities for their future students. They also point out the motivation and interest generated by the task, as well as the fact that visually supported storeys are easier for English learners to understand.

In another case study with a sample of 30 students from Kyrgyzstan aged between 12 and 16 years, Chubko et al. (2020) used digital storytelling in science with non-native English students. This study is an extension of a previous study, the Indigenous Sky Stories program, conducted with Australian primary school students aged 10–12 years (Ruddell et al., 2016). The conclusions reached in Chubko et al. (2020) are that the creation of digital narratives promotes the literacy of scientific concepts, both in students who master the language of instruction, and in those who do not have this mastery, as demonstrated in an extensive case study, consisting of a sample of over 300 Australian and Kyrgyz students. The authors claim that the use of digital storytelling promoted creativity in constructing a digital narrative and the processes involved in this construction reduced the gap between native and non-native English students.

Naqvi and Al Mahrooqi (2016) discuss an experience with Omani university ESL students. These students were divided into two equal groups: one group of students collaboratively created a digital video showing an audiovisual message in English about a commercial product; a second group, based on this video, carried out a collaborative writing exercise in English on the form and content of the video. After carrying out this exercise in different work sessions, the researchers of this study designed a questionnaire to analyse the learners’ impressions of learning English as an L2 using digital videos and collaboratively written reports on these videos. The results obtained showed positive impressions of the group of learners related to the good use of digital tools in collaborative and creative environments for ESL students.

Comas-Quinn et al. (2009) carried out a study with a group of English college students of the Open University of how mobile phones favour spatial mobility for language learning. In this way, from a shared blog, the mobile device becomes a tool for capturing inputs of any kind related to linguistic and cultural structures of an L2 which can then be shared among peers in a blog. The way to share them is through the creation of digital texts and narratives.

#### Technology as a Tool That Develops Linguistic Thinking

Different studies claim that interactive technology features related with the co-presence, in one single space of multiple different perspectives, stimulates further thinking. In this space, students can make their ideas visible, externalise their thoughts and represent ideas using multimedia and multimodal facilities. These features of technology can support the generation of new ideas, the connection between seemingly disparate bits of information from divergent perspectives and the construction of a holistic view of the information involved. As a result, different studies claim that the use of technology to solve linguistic challenges co-creatively can develop variables related to linguistic thinking. This is related with the notion of thinking creatively in terms of ‘we’ and the cultural back as the central axis around which novel ideas are generated and a viable approach for addressing creativity as a culturally diverse capacity (Tanggaard, 2020).

In this line, Ntelioglou et al. (2014) carried out a case study in an inner city elementary school with a large population of recently arrived and Canadian-born linguistically and culturally diverse students from Gambian, Indian, Mexican, Sri Lankan, Tibetan and Vietnamese backgrounds, as well as a recent wave of students from Hungary. The study reports how the use of creative digital tools, such as iMovie and iPhoto for writing descriptive texts, had a positive impact on the expression of personal identity. The texts written by the students included photographs of the selected spaces, descriptions, emotions and experiences of the students in these places. These authors also pointed out that the texts were of very good linguistic quality. Therefore, the students learnt a basic competence in language learning: the written expression of the language. Moreover, the use of technological tools changed the dynamics of learning in the classroom towards more participatory learning processes that included aspects of self-identity and emotions. Therefore, instead of promoting quickly installed functional thinking skills, uniformly defined across cultures, technology promoted creativity as it is a kind of agency in the world, differently defined in various contexts because these require us to act in different creative ways according to the circumstances (Glăveanu et al., 2016).

Lee (2009) describes how through collaborative blogging and collaborative podcasting, university students, from America and Spain, developed their communication and cultural awareness. The blogs and podcasts created were exchanged between the two cultures with a view to offering and receiving feedback for language correctness. The students did not receive prompts on how they should offer feedback, but instead made their own decisions. At the end of the study, students highlighted that they would not have participated in interactive discussions on linguistic and cultural aspects if they had face-to-face meetings.

Tyrou’s (2021) involves 92 university students of Italian as a foreign language in a Wiki environment with a series of activities that encouraged creative thinking, such as visiting virtual museums, and then writing texts collaboratively. The study analysed collaborative writing using Wiki tools in second language teaching. The use of Web 2.0 tools promoted improved learning processes through participation, collaboration and teamwork. This type of collaborative writing mainly improved the process of text revision, favouring both self-correction and peer correction. After analysing student perceptions about web 2.0 technologies for language learning, Tyrou (2021) concludes that ‘online collaborative wikis tools can increase knowledge of culture and foreign language, promote teamwork and familiarise our students with new technologies and virtual museums’ (p. 53). These are recognised creative values capable to develop students’ creative capacity (Tanggaard, 2020).

In this line of work, Mellati and Khademi (2015) explore the possibilities of the WhatsApp mobile application as a tool that favours the sense of belonging to a group. The experience was carried out among 68 Iranian students of English aged between 18 and 35, with an intermediate level of English in the context of a course called Online Mobile Language Learning Course. The experience, in terms of collaborative and creative writing, was very positive. The only drawback observed was that, as the course progressed, a more careless use of the language was observed.

Yang and Yeh (2021) proposed the use of YouTube for teaching and learning the socio-cultural component of the English language. The research was carried out among 71 university students who wanted to learn English. In addition to making videos that were later posted on YouTube, this activity was followed by a critical reflection on the audiovisual production made, in order to reflect with the class group on the socio-cultural component that they wanted to transmit.

Also related to writing texts, Mellati and Khademi (2014) reports on the impact of peer assessment in a technology-based language environment on the quality of creative writing and the development of writing skills. In this study, so-called E-collaboration emerges as a highly intrinsically motivated pathway, as cooperative tasks, specifically based in digital environments, lead to the development of group work and communal learning that positively redounds to individual learning. The results of this research showed that peer learning through Computer-Assisted Language Learning can not only facilitate the development of language skills related to writing texts but also enhance intercultural communicative competence and digital literacy, understood as the ability to locate, organise, understand, evaluate and analyse information using digital technology.

### Technology as a Medium That Facilitates an Appropriate Context for Co-creation in Language Education

Dynamic and multimodal interaction within a technology environment affords unique opportunities for learners to co-create in language education. Digital technologies can create rich and resourceful environments capable of acting as a medium which stimulates, orchestrates and supports specific creative processes. Sun et al. (2022) claim that the features of digital technologies can enhance key creative processes, such as emerging of new ideas, identifying connections between seemingly disparate bits of information, fostering collaborations, elaborating the information and promoting imaginative expressions.

The qualitative analysis of the papers selected for this review distinguished three different uses of digital technologies as medium for co-creation: (a) building an immersive and creative experience by providing a wide range of technologies; (b) the use of dedicated technology for building a co-creative writing community and (c) orchestrate the collaborative creativity process. Next, we address the discussion of the results obtained in relation to these two uses of technology as a medium for co-creation.

#### Building an Immersive and Creative Experience by Providing a Wide Range of Technologies

Technology plays a crucial role when it comes to developing creativity and creative learning environments for second language acquisition. As Lorenzo et al. (2013, p. 1615) state, such an environment ‘promotes an immersive, creative and collaborative experience in the process of learning a foreign language’. These virtual learning universes can change the nature of teaching by simultaneously providing a social, immersive and creative experience for second language learners (Canfield, 2008; Chan, 2008; Cooke-Plagwitz, 2008; Jeffery and Collins, 2008).

The purpose of the study by Lorenzo et al. (2013) is the creation of a *Massively Multiuser Online Learning (MMOL)* in university classroom, a didactic strategy that makes use of ICT to improve learning processes in a group of students in face-to-face mode. This integrated platform for massively multiuser learning allows the creation, development and deployment of content and activities for teaching a language in a virtual world. The cooperative, collaborative and socially interactive nature of students as well as teachers is based on a 3D online education environment, which in turn is supported using microcontent immersed in collaborative virtual environments. In other words, the microcontent identified in one of the microformats recognised by the *MMOL* tool is the basic unit of these environments. The *MMOL* microformat may be the same as the one used in Web 2.0, but its meta-description requires further improvement so that it can be intensively reused in any virtualised scenario, or, failing that, adapted to the conditions of a specific context.

The results of this action research with the use of *MMOL* for second language learning show that the possibility to cooperate and collaborate in a 3D educational context, in combination with the use of communication tools (e.g., chat, video chat or VoIP) and intelligent assistants (chatbots or NPCs), help the learner to accept a role of acceptance and objective criticism for group learning, which has a positive effect on individual acquisition of second language linguistic content.

A specific use of audio and video platforms is the *Xtranormal* environment (Kilickaya, 2010) which allows the creation of animated films with voice audio. The creation of digital products of this type, in an L2 teaching and learning university environment, favours the creation of 3D characters playing different roles for language learning. In this way, listening contexts are generated to improve writing, reading and, in particular, the pronunciation of a second language.

Olivier (2019) explored how the creation of videos can be used to motivate students to interact critically with digital content and participate collaboratively using new technologies in learning a language. The experience was carried out among 82 university students, who produced a total of 50 multimodal creations individually, in pairs or triads. Some of these creations consisted in animations created online, others were animations made with PowerPoint with voice-overs, although they referred to all of these with the umbrella term ‘video’. The recorded videos are short videos with the purpose of being open educational resources, on topics provided by the teacher, all related to language learning. No instructions were given to the students on how to plan, write the script or shoot the videos. The conclusions of this research show that the students had to face difficulties not so much related to the use of technology, but rather content selection and condensing information, since the videos were limited in duration. In the same way, the students became real actors in this teaching and learning process, and valued the use of technology as a means of encouraging creativity, as this methodology broke away from traditional practices in language teaching and learning.

Akinwamide and Adedara (2012) designed a platform that provided different digital tools to help teachers working at different levels of teaching and learning digitalize the teaching and learning of a language. Finally, the article by Anderson et al. (2018) presents the findings of a global literacy project based on digital storytelling. They work on multiliteracy through a methodology based collaborative and dialogic ways, allowing for the sharing of divergent thoughts in each community. This research demonstrates the importance of an integrated and inclusive approach to languages in the framework of multiliteracy. Authors conclude that multimodal storytelling develops creative and dialogic thinking.

#### Dedicated Technology for Building a Co-creative Writing Community

Mak and Coniam (2008) recommended the use of the wiki for language learning. In their study, they used the wiki as an online, co-creative and multimedia environment for writing in English (ESL) with 11-year-old students from Hong Kong, who were not used to working collaboratively. In groups of four students, they participated in a project aiming to describe the facilities and characteristics of their educational centre to create an advertising brochure for promoting the centre. As the project progressed, the text of the group under analysis improved its quality and complexity. In addition, by writing a collaborative text, the students learned to expand, reorganise and correct their own writing and their group mates’ writing. The study highlights that the brochure included creative and original multimedia information.

Lund and Rasmussen (2008) also highlight the use of the wiki as a collaborative and creative technology with high school students from a Norwegian institute who participated in a collaborative writing project to describe a typical English city, within the framework of the subject of ESL. The creative component of this activity was found in the invention of a city based on the real characteristics of British cities using both textual description and images. The students undertook the presentation of their wiki, making use of their imagination. During the development of the project, the students interpreted, constructed and reconstructed writing processes. They went through each process as a result of the following actions: reviewing the wikis of the other groups and becoming aware of the global work of all their colleagues; adapting their texts; and coordinating with the members of their own groups to divide their workload. This led them to create a group task identity and commit them to the task.

Armstrong and Retterer (2008) investigated how the use of the Blog influences foreign language learning (in this case, students of Spanish, of unspecified ages, with an intermediate level of Spanish). Two different activities were planned as: (1) writing a storey among the whole group-class; (2) writing several personal blog posts for each small group. For the first activity, the students created a storey together, over the course of 3 weeks. The teacher started the storey and the different groups of students continued to build it on the basis of the following instruction: each group had to add information to the storey twice a week, but not on the same day, so they had to read the contributions of their peers. In the end, they recorded the storey as if it were a movie. As a result of the study, students who wrote on the blog using a significant number of words, improved their oral expression in terms of accuracy of verb tenses and also increased the complexity of their sentences. An anonymous questionnaire to the students about their blog writing experience showed that 100% of students felt more comfortable writing in Spanish at the end of that experience.

Rojas-Drummond et al. (2008) focused on 6–9-year-old students in Mexico City and how they learned collaboratively in creative writing projects through the use of ICTs. They started from a working context that adopted the model of a learning community. This promoted the social construction of knowledge among all participants. The construction of texts and multimedia products of storeys created by groups of children from fourth to sixth grade, through the innovative educational programme *Learning Together*, revealed the dynamic functioning in educational environments of some central socio-cultural concepts. Thus, collaborative creativity came across in the writing of texts that involved co-construction of texts; the establishment of intertextual and intercontextual relationships between the texts themselves using ICT; the development of dialogic and textual production strategies; and the appropriation of diverse cultural artefacts for the construction of knowledge.

#### Orchestrate the Collaborative Creativity Process in Language Education

Schmoelz (2018) reports how secondary education students who use digital narratives to encourage co-creativity show a greater commitment, at the time of planning the writing activity and a high control and effectiveness in the development and resolution of the activity. In the digital storytelling phase, students experience enjoyment and fun that allows a better-constructed storeys. The qualitative study covers 125 students who are interviewed, questioned, recorded and discussed.

## Conclusion

This paper reviews studies of designs of technology-enhanced learning environments that promote collaborative creativity skills in language education. The final objective of this review has been to capture advanced knowledge for designing future language technology-enhanced learning projects capable of promoting key collaborative and creative processes.

This paper aims to fill a gap in educational research around the use of digital technology to promote collaborative creativity skills. Our selection criteria include four essential research variables to enhance creativity in a global knowledge society: collaboration, creativity, technology and language education. Only 26 studies meet all these criteria.

Although digital and interactive technologies are claimed to create a favourable language learning environment capable of fostering creative and collaborative language learning and writing (Wang and Vásquez, 2012), most of the studies reviewed have not been explicitly designed to improve and evaluate creativity as a social and collaborative endeavour. On the contrary, the importance of creativity in these papers is limited to the creation of a purely digital linguistic product, such as a text, a video or a podcast (Andayani, 2019). Therefore, this review paper can be taken as the basis for future research in language education.

From our review study, we conclude, firstly, that the features of digital and interactive technologies enable the design of powerful and rich language learning environments for knowledge co-creation. These technology-enhanced learning environments open up new opportunities for learners to, collaboratively, generate, modify and evaluate new ideas through online and multimodal interaction.

Secondly, the qualitative analyses of the selected papers conclude that technology can play three important roles to favour co-creativity in language education, namely, tutor, tool and medium. Technology can act as a tutoring device that guides the implementation of key co-creation skills. Therefore, there is a pattern of work and action that leads to solving language problems with digital tools that promote collaborative work, albeit in a sequenced way: Blogs, Wikis, WebQuest, Kahoot and YouTube as the most popular environments (Contreras Salas, 2012).

Besides, technology can act as a tool that enables and shapes the development of co-creative thinking skills. Therefore, creative thinking arises from the use of technology that shapes the thinking of its users. This is where creative writing of digital narratives in environments, such as IMOvie, Iphoto, TOEFL Writing Test (Mellati and Khademi, 2014) or *MMOL* for second language learning emerged.

Furthermore, technology can play the role of the medium that creates rich and resourceful environments to stimulate the emergence of collective creative processes. From this point of view, blogging (Armstrong and Retterer, 2008) or the use of Wikis as online collaborative writing environments (Mak and Coniam, 2008) allows students to improve their co-written writing skills by building texts in digital environments and encouraging e-collaboration between them.

Thirdly, six different forms of technologies have been identified in the reviewed studies that promote co-creativity in language education. They are the following: audio and video platforms, web-based environments, wikis, mobile technology and word processors. These forms of technology support online group learning that enthral students in active and resourceful-user experience for collaborative knowledge creation.

Finally, our work has its limitations that may have conditioned our results because of having discarded papers that could have contributed to answering our research question. Among these limitations, we highlight the following three: (a) limitation in the type of publications considered: only articles that followed a blind peer review procedure were considered; (b) limitation in the language chosen: only articles written in English and Spanish were included; and (c) limitation in the search keyword strategy and that these could be insufficient to include key articles in our field of study. However, in an attempt to minimise these limitations, firstly, a systematic review methodology was followed. Secondly, the most significant and prestigious databases in the field of education were consulted: Web of Science, Scopus and Google Scholar.

As a final remark, this paper gives evidence of how technology can support the learning of key linguistic and literary processes, such as: speaking, listening, reading and writing. Furthermore, this paper concludes that co-creativity is an intrinsic phenomenon of literary knowledge. However, there is a need to develop future language technology-enhanced learning projects capable of promoting key collaborative and creative processes in language education. We hope this paper may contribute to reaching this objective.

## Data Availability Statement

The raw data supporting the conclusions of this article will be made available by the authors, without undue reservation.

## Author Contributions

All authors listed have made a substantial, direct and intellectual contribution to the work, and approved it for publication.

## Funding

This research has been funded by the Ministry of Science and Innovation of the Government of Spain under Grant EDU2019-107399RB-I00.

## Conflict of Interest

The authors declare that the research was conducted in the absence of any commercial or financial relationships that could be construed as a potential conflict of interest.

## Publisher’s Note

All claims expressed in this article are solely those of the authors and do not necessarily represent those of their affiliated organizations, or those of the publisher, the editors and the reviewers. Any product that may be evaluated in this article, or claim that may be made by its manufacturer, is not guaranteed or endorsed by the publisher.
